# A Wide-Range, Wireless Wearable Inertial Motion Sensing System for Capturing Fast Athletic Biomechanics in Overhead Pitching

**DOI:** 10.3390/s19173637

**Published:** 2019-08-21

**Authors:** Michael Lapinski, Carolina Brum Medeiros, Donna Moxley Scarborough, Eric Berkson, Thomas J. Gill, Thomas Kepple, Joseph A. Paradiso

**Affiliations:** 1 Responsive Environments Group, MIT Media Lab, Cambridge, MA 02139, USA; 2 Input Devices & Musical Interaction Lab, McGill University, Montreal, QC H3A 1E3, Canada; 3 Sports Medicine Service, Department of Orthopaedic Surgery, Massachusetts General Hospital, Boston, MA 02114, USA; 4 New England Baptist Hospital, Boston, MA 02120, USA; 5 C-Motion Inc., Germantown, MD 20874, USA

**Keywords:** baseball, pitching, ballistic motion, jerk, wearable wireless sensor, high-dynamic range motion capture, wearable inertial sensor, wearable IMU, wireless wearable motion sensing, MARG, inertial measurement vs. optical tracking

## Abstract

The standard technology used to capture motion for biomechanical analysis in sports has employed marker-based optical systems. While these systems are excellent at providing positional information, they suffer from a limited ability to accurately provide fundamental quantities such as velocity and acceleration (hence forces and torques) during high-speed motion typical of many sports. Conventional optical systems require considerable setup time, can exhibit sensitivity to extraneous light, and generally sample too slowly to accurately capture extreme bursts of athletic activity. In recent years, wireless wearable sensors have begun to penetrate devices used in sports performance assessment, offering potential solutions to these limitations. This article, after determining pressing problems in sports that such sensors could solve and surveying the state-of-the-art in wearable motion capture for sports, presents a wearable dual-range inertial and magnetic sensor platform that we developed to enable an end-to-end investigation of high-level, very wide dynamic-range biomechanical parameters. We tested our system on collegiate and elite baseball pitchers, and have derived and measured metrics to glean insight into performance-relevant motion. As this was, we believe, the first ultra-wide-range wireless multipoint and multimodal inertial and magnetic sensor array to be used on elite baseball pitchers, we trace its development, present some of our results, and discuss limitations in accuracy from factors such as soft-tissue artifacts encountered with extreme motion. In addition, we discuss new metric opportunities brought by our systems that may be relevant for the assessment of micro-trauma in baseball.

## 1. Introduction

Elbow and shoulder injuries among baseball players, in particular pitchers, continue to be a concern despite maximum pitch count recommendations and regulations [1,2,3,4,5,6]. Ligament and muscular damage at the elbow and shoulder has been associated with the repeated micro trauma sustained by these structures during the demands of high-speed throwing and pitching [1,7,8]. In addition to financial costs associated with ligamentous injuries, typically requiring surgical treatment, functional day-to-day limitations and a long rehabilitation process create further loss to an athlete and/or an athlete’s professional organization. Measurements of accelerations and angular velocities per segment, plus computed torques and forces on the joints during pitching, may lead to better development of injury avoidance and return to sport after injury programs. Currently, optical motion capture is the standard tool that sports medicine biomechanists and clinicians use to study the mechanics of motion and their correlation with injuries. These systems provide data to guide diagnosis, treatment, training modifications, return to sport, or removal from training.

New technology is advancing motion capture to wearable sensor systems. The quality of these systems ranges from gadgets with limited or no calibration to accurate scientific tools. Whether lab-based or body-worn, current technologies are limited by sampling body segment motion collection at rates too slow to fully capture the ballistic human motion performed during pitching. The act of pitching includes body segment motion which is relatively slow at the start of the activity, creating a base for transferring momentum through the body in a proximal-to-distal pattern out to the throwing arm [9,10]. Pitching also includes the fastest recorded human body segment movement; the arm segment rotating about the shoulder joint measuring over 7000°/s [11]. In recent years, physicians and team managers have observed, with attention and enthusiasm, the possibilities brought by new technology and methods for quantifying and qualifying high-speed sport performance [12,13,14]. Using systems that evolved from our initial prototypes fielded in 2006, this paper reports one of the earliest efforts, to our knowledge, of providing reliable sports data using portable wireless wearable electronics that leverage an ultra-wide-range wireless multipoint inertial and magnetic sensor array.

As introduced above, most quantitative athletic biomechanical analyses still rely on manual video inspection or commercial marker-based optical systems [15,16], which consist of near-infrared camera arrays measuring at up to hundreds of frames per second, compromising resolution for capture speed. Setting up and calibrating a camera-based tracking system is time consuming, and the stability of the data processing can be affected by visual artifacts, occlusion, and changing background light. The expenses to purchase, maintain, and operate camera-based laboratories also limit access to biomechanical pitch analyses for many institutions and athletes. The accepted standard motion capture systems are mostly indoor lab-based equipment setups, which limit simulation of the outdoor game environment and potentially the athlete’s performance. Commodity depth-sensing cameras, as embodied in the Microsoft Kinect™, have had some application in sports analysis [17], but range, speed, and accuracy limitations have constrained their capability. Active magnetic trackers are light-insensitive, but susceptible to distortion from conductive and/or ferrous metal and present very limited range of operation, as well as often inclusive of tethered cabled sensors [18]. Mechanical measurement methods, such as goniometers [19] and exoskeletons [20], require the body to be restrictively cabled up or constrained. Vests, shirts, and garments, generally wired with embedded inertial, bioelectric, and fabric sensors, have likewise been explored and adapted for motion capture, including athletic sensing [21,22,23,24,25]. One example is a system composed of a single inertial sensor applied onto the elbow via a skin-tight sleeve [26,27] for athletic applications. Also, high-quality flexible goniometers with embedded inertial units have recently become available [28].

Sensors of nearly all types have grown smaller and cheaper, enabling their seamless integration into nearly everything, as envisioned decades ago by the pioneers of Ubiquitous Computing [29]. Inertial systems have a limited history in basic motion and biomechanical research, dating back to the 1970s [30], before integrated miniature accelerometers were available. Wired and wireless wearable inertial systems have appeared commercially over the last decade (e.g., [24,25,26,28,31]) and in research (e.g., [32,33,34,35]), but have been mainly applied to non-ballistic motion capture, where the average motion speed is typical of human gait, as opposed to high-intensity sports analysis, only very recently providing the ability to capture high speed motion [36]. Some researchers use inertial technology to only recognize posture and activity, dispensing the need for high range sensors and joint angle computation [37], albeit at an information sacrifice. Additional information can be found in these recent review articles discussing the use of inertial sensors for lower limb movement [38,39], generic human motion [40], and sports [41].

However, while the product market has been successful in putting these small wearable devices on athletes and moving the athlete out of the lab setting, the data application in sport is still constrained by range and sampling rate [42]. To address the challenge of quantifying the high-speed stresses incurred on the upper extremity during throwing, specifically baseball pitching, we set out to create a new inertial measurement unit (IMU) that can capture 3D motions occurring at both low and high speeds. Accordingly, we have developed a wearable inertial sensor platform to enable end-to-end investigation of high-level, very wide dynamic-range biomechanical parameters. Unlike commercially available wireless systems that have been designed for motion capture, our device has extremely high dynamic range and exhibits precise synchronization across multiple wearable nodes.

Using the state-of-the-art camera-based motion capture systems, shoulder and elbow distraction forces are calculated using the second derivative of the measurement system data, i.e., linear acceleration, and inverse kinematics. Unfortunately, the derivatives of orders greater or equal to two have high levels of noise, often resulting in limited or no physical significance, unless the original data—in position units—is filtered down to 10–20 Hz. This filtering damps rapid signal variations, and hampers proper inference of higher-order derivatives that happen during excessive joint load. Accordingly, we assert that classical optical systems are limited in producing meaningful assessment of these forces.

Finally, we introduce the concept of jerk to the evaluation of pitching mechanics using our IMU system. As defined in classical mechanics literature (e.g., [43]), jerk is the third derivative of position, and it expresses the rate of change of acceleration (as opposed to acceleration itself which is the rate of change in velocity over time). We suggest that the rate of change of acceleration may be more related to microtrauma than the absolute value of acceleration, which is canonically used to obtain force metrics. Given the assertion about the noise inherent in the second derivative of the positional data to calculate acceleration, calculating a third derivative of positional data has been effectively prohibited in previous optical-based biomechanical evaluations of pitching. We hypothesize that meaningful jerk data could be obtained from our multi-segment inertial system.

In this paper, we present the scientific requirements needed and the steps taken to build a robust and accurate wearable sensing system with high autonomy and portability for baseball and provide initial comparisons to an optical motion analysis system. In baseball, pitch type is often distinguished based on the grip of the baseball and the motion of the hand and forearm. Wrist flexion and extension rely on the action of the larger muscles in the forearm, some of which cross the elbow joint. Therefore, we included wrist joint force and hand angular velocity in our analyses. Elbow valgus/varus torque, and shoulder and elbow distraction forces were biomechanical metrics selected for comparison based on their established connection to shoulder injuries and UCL (Ulnar Collateral Ligament) sprain [44,45]. A series of studies were performed to address the following aims: (1) Compare the raw output of wrist force, wrist angular velocity, shoulder angular velocity, and shoulder and elbow distraction forces between an optical marker-based system versus our developed inertial system. (2) Investigate the influence of filter processing on optical system data compared to the data from the multimodal wide-range IMUs. (3) Investigate the feasibility of using shoulder jerk as a metric from the IMU wearable system for identifying differences in stress at the shoulder joint across pitch types.

## 2. Materials and Methods

### 2.1. Participants

**Figure 1:**
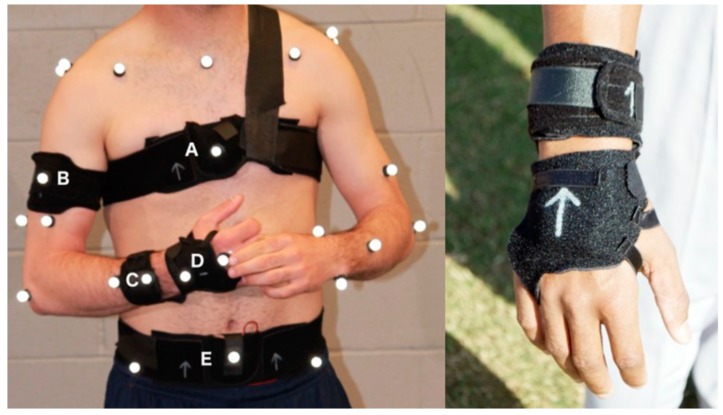
Neoprene straps worn by to a pitcher (**left**), node locations: chest (A), upper arm (B), forearm (C), hand (D), waist (E). Detail of forearm and hand nodes (**right**).

This pilot study was approved by the institutional review board and included two sub-studies. In the first study, we collected simultaneous data from an optical 3D motion capture system in our Sports Performance Laboratory and our multi-segment inertial system on two collegiate (age 20.5 years) pitchers. A second data set was collected on six professional baseball pitchers using our multi-segment inertial system at an outdoor training facility. All participants provided informed written consent. Both studies included placement of five multi-segment inertial measurement units (nodes) to the wrist, the forearm, the upper arm, the chest and the waist on each participant (Figure 1). In order to affix the sensors to the players, we co-designed with an orthotics manufacturer, a set of rubberized Neoprene Snakeskin™ straps with snug pockets to securely hold the sensor nodes [46]. Among the five pockets, only the one placed on the chest required additional straps to keep it in place during fast motions (Figure 1, node A). Due to sweat and the fast motion, some of the optical system’s reflective spherical markers came unfixed from the throwing arm. In order to replace them at the same anatomical point, we proactively labeled the skin with an ink pen prior to marker placement.

The testing procedure for both studies included a warm-up routine prior to data collection, after the nodes (and reflective markers) were applied. All participants threw the full distance of 18.44 m from a standard turf mound to a target placed approximately 1 m behind a standard sized home plate. A professional grade radar gun, Stalker ATS 5.0 (Stalker Radar, Plano, TX, USA), was used to record all pitch speeds by measuring the velocity of the ball along the radar’s line of sight using standard Doppler techniques. Players pitched their standard ‘side’, throwing a minimum of 25 pitches with a mix of fastballs, breaking pitches and change-ups. Pitch Type was recorded. Each subject’s set up time included approximately 15 min to prep the skin (place ink marks where the markers were to be placed on the throwing arm) followed by the reflective marker placement. A 10-min time was allocated to place the IMUs and collect calibration information of their positions. Depending on the pace of the pitcher and number of pitches thrown, the data collection time was approximately 20 min. The removal of the sensors took about 5 min, resulting in a total study time of approximately 1 to 1 and a quarter-hours in length. All results provided in this paper refer to Study 1, except the jerk analysis.

### 2.2. Wearable Sensor Hardware and Software

**Figure 2:**
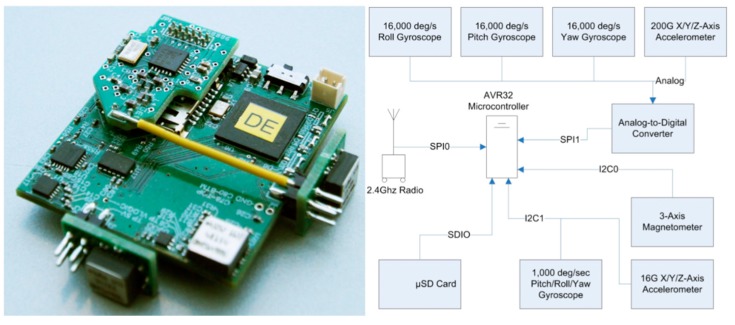
Final Wearable ‘Sportsemble’ Sensor Node (**left**) and Block Diagram (**right**).

Our system had its genesis in a multipoint wearable inertial sensor network that we originally designed in 2006 to instrument an interactive dance ensemble [47]. This system was evolved from a wireless multimodal sensor node that our research group designed and first fielded in a dancing shoe in 1997 [48], which was subsequently adapted into a very early sensor node for wireless gait analysis [49]. From 2006 to 2013, we developed a succession of devices aimed at pitching and batting [50,51,52], each honed by experience garnered in working with professional players during spring training, resulting in our final design shown in Figure 2 and detailed in [46].

The nodes measure (45 mm × 50 mm × 10 mm) and weigh 25 g (half due to battery mass). Each node has a 3-axis ± 200 g ADXL377 accelerometer, three orthogonal single-axis, ±20,000°/s ADRXS649 gyros, a 3-axis Invensense IMU-3000 3-axis 1000°/s gyro, a 3-axis ± 16 G ADXL345 accelerometer, and a HMC5843 digital magnetometer (Figure 2). The multi-range accelerometers and gyroscopes let us record slow motion with the low-range devices and fast motion with the high-range units, thus providing high relative resolution across an entire athletic gesture after being fused in a statistically-based postprocessing interpolation [46]. Synchronized inertial data was sampled at a rate of 1000 Hz across all calibration and pitch gestures.

The nodes are supervised by an AVR32 processor with a removable SD memory card used to store all data locally (these are removed and uploaded to mass storage at the end of each day). The embedded radio, a 2.4 GHz Nordic nRF2401a with RF amplifier, provides a maximum bandwidth of 1 Mbps at an output power of +4 dBm. It is mounted on a daughter card to isolate the RF electronics from the main board and enable an easy upgrade of the RF hardware. Our radio protocol is a custom-designed lightweight TDMA (Time-Division Multiple Access) scheme [53] and is primarily used to synchronize all nodes to <1 ms, demark different test runs, and monitor diagnostic information that is indicative of node health. Our nodes use a 145 mAh lithium polymer rechargeable battery that can continuously power a node for circa 3 h of use. The nodes are continuously active when switched on—although adaptive power management techniques can reduce the average needed current, this degree of node longevity is adequate for our typical testing session. As the inertial components come with inexact specification, each sensor on each node is custom-calibrated on a controlled highly accurate rotating platform [46].

### 2.3. Optical 3D Motion Capture System Hardware and Software

A Vicon MX™ 3D motion capture system (Vicon Motion Systems Ltd., Oxford, Oxfordshire, UK) comprised of 20 T-series cameras (collecting at 360 Hz) was used to track the 62 reflective markers (14 mm diameter spheres) placed upon each pitcher during the pitching motion. The markers were located over anatomical landmarks to identify joints, and additional markers were placed in general locations upon each segment to improve segment tracking in the 3D space. These specific marker placements were based on a previously described marker set [10] that creates a 15 body-segment model for data capture during baseball pitching. For global coordinates, the direction of the pitcher’s throw defined the motion capture laboratory’s X axis. The Z axis was identified as the vertical direction, and Y defined as the cross-product of the X and Z axes. The C3D motion capture data files were imported into a biomechanical analysis software, Visual3D™ (Version 5, C-Motion Research Biomechanics, Inc., Germantown, MD, USA) for joint torque and force calculations. The methodology that Visual3D uses for computing kinetics is described in detail in the literature [54]. Our contribution in relation to the cited work is that the angular velocity and the translational acceleration data used in the computations is taken directly from the sensors, instead of being estimated from the optical data. All joint torques were calculated with respect to participants’ height. Particular details to note for these study comparisons include the determination of the shoulder joint center following linear regressions described by Meskers’ protocol [55]. The local coordinate systems for each node location are defined in [46]. In the case of the hand and arm mounted IMUs and optical targets described here, the local Z_a_ axis of the arm segment was aligned with the longitudinal axis of the humerus (and the local Z_h_ axis of the hand pointed down along the arm) to create 6-DOF models of the hand and arm segments. Our modeling of the upper extremity joint followed recommendations of the ISB [56].

A hardware interface allowed synchronization between the wearable multimodal IMU system and the optical motion capture system via a general-purpose input output (GPIO) port on the base station transmitter. The synchronization data was stored on each node’s onboard storage and was rectified in the analysis engine to provide a single synchronized data set between all nodes and the optical system.

The basestation is used for time synchronization of the nodes, and it does not process any of the data. The basestation sends timestamped synchronization packets and commands to the nodes. When a node receives these timestamp packets, it annotates the data that was being gathered in real-time with the timestamp. This made data alignment between the nodes fairly trivial, as all that was required to align the data was to match timestamp values [46,53].

### 2.4. Data Analysis

It is common practice in the field of biomechanics to filter out data derived from the optical systems [10,45,57,58,59,60,61], as smoothing positional data allows for the use of the optical data for computing kinetic quantities. A qualitative comparison of optical and inertial data was performed with and without canonical low pass filters on optical data. A fourth-order, a low-pass Butterworth filter was applied to the data from the optical marker set at 14 Hz. Attenuation between the optical and the inertial systems were calculated for the kinetic and kinematic variables of interest.

Measurements of node placement on each body segment were made relative to anatomical features [46] and permitted translation between coordinate systems. Kinematic and kinetics data, including shoulder internal/external rotation torques, elbow valgus torque, and shoulder and elbow distractive forces, were calculated for both systems. In the second evaluation, pitch motions were evaluated with the inertial system alone.

**Figure 3:**
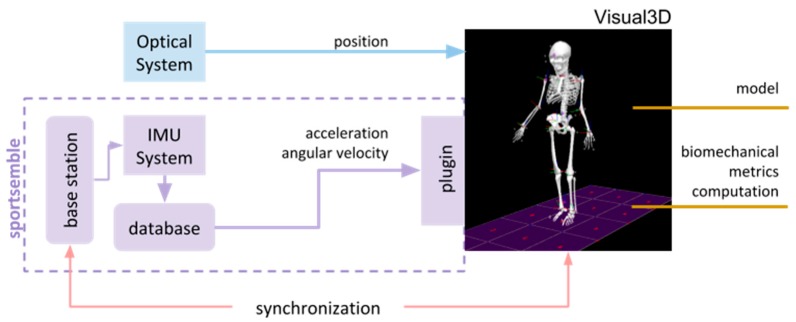
System architecture merging optical and inertial data for kinetics and dynamics processing.

We used the same biomechanical analysis engine, Visual3D™ (v5) [62], to standardize calculations of kinematic and kinetic data from both the IMU and the optical motion capture systems. This allowed for direct comparisons between the two data collection approaches (Figure 3). The IMU data undergo minimal processing—we essentially input the directly-measured angular rates and accelerations into Visual3D at the node locations instead of using the analogous values that would be derived by complex analysis of the optical markers. The synchronization time stamp data was stored on each node and rectified in the analysis engine to provide a single synchronized data set between all nodes and with the optical system.

A series of descriptive analyses are presented in this proof of concept study. The limited number of participants in the simultaneous biomechanical comparison study limited the application of deeper statistical analyses.

## 3. Results

To meet the aims of this study, we collected data in two separate testing scenarios. We present first the results of the biomechanical metrics of both the multimodal IMU system and the optical system during the simultaneous data capture testing. The two collegiate pitchers in study one included Participant A (height = 1.78 M and weight 82.7 kg) with an average fastball speed of 124.4 km/h. Participant B (height of 1.80 M and weight 79.1 kg) performed during testing an average fastball speed of 114.9 km/h.

### 3.1. Qualitative Comparison of Data from the Multimodal IMU System and the Optical System

**Figure 4:**
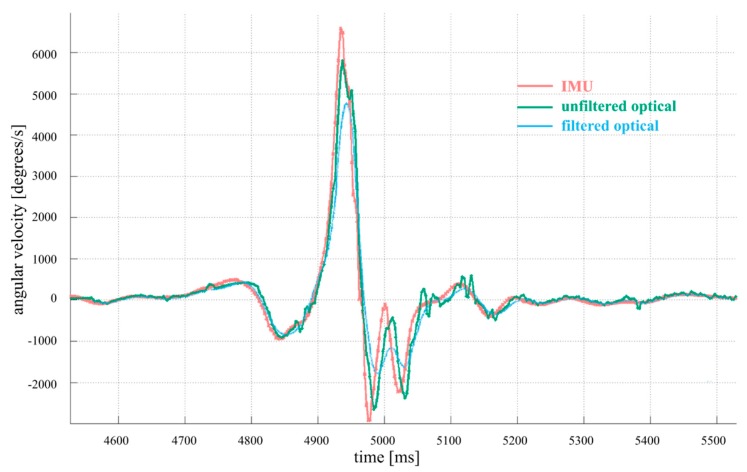
Z_h_ axis angular velocity of the hand for a typical fastball pitch: IMU, filtered and unfiltered optical data. The loss of information on filtered optical data is noticeable in the amplitude and dynamics.

The final velocity provided to the gripping fingers just prior to ball release is generated by the hand segment of the pitcher’s throwing arm. The angular velocity of the hand along axis Z_h_, illustrates the timing lag and peak attenuation introduced by the standard low pass filtering of the optical motion capture system (Figure 4). The use of unfiltered optical data mitigates this effect, resulting in a more similar optical data motion pattern to that of the multimodal IMU system. Averaging across 26 throws for one of the pitcher subjects, we saw a mean difference between canonically filtered optical and inertial angular velocity distractive (positive) peaks at the hand along the (Z_h_) axis of 32% (2071°/s out of 6466°/s), consistent with the example shown in Figure 4. The standard deviation in this inertial peak across all throws was found to be 331°/s (5% of the average peak angular rate) and the standard deviation in the inertial-to-optical difference in angular velocity was 491°/s (24% of the mean), indicating a consistent undershoot in the optical measurement. The Z_h_ axis considered here at the location of the hand involves some of the highest and most impulsive angular rates encountered in the throwing motion, as it corresponds to wrist/arm twist. Nodes at other body locations and along other axes exhibit lower peak rates, although still tend to exhibit optical-to-inertial undershoot because of fast-changing dynamics that the inertial system detects and optical system misses [46]. Further statistical discussion is given in Section 3.2.

**Figure 5:**
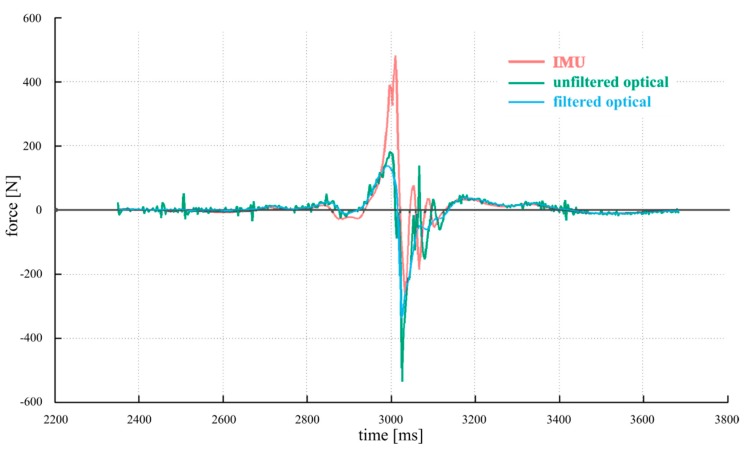
Sagittal plane X axis (throwing direction) wrist joint force for a typical fastball pitch: IMU, filtered and unfiltered optical data.

In Figure 5, we present comparisons between the IMU data and filtered and unfiltered optical data for a measurement of the sagittal plane X axis wrist force (along the direction of throw). As anticipated, the unfiltered data exhibits considerably more noise than either filtered or IMU data. During the joint compression force phase (positive values of force), however, both filtered and unfiltered optical data are not able to capture the peak of the motion seen by the IMU, falling over a factor of two short in this axis, indicating a serious underestimate in the most critical phase of pitching motion.

After the ball release, high speed video has shown that several apparently involuntary and somewhat oscillatory movements happen in the kinematic chain [63] as the relaxing arm rocks back and forth, contributing to the ringing seen in our data there in both Figure 4 and Figure 5. What aspects of these motions are actually biomechanically relevant motion or arise from soft tissue artifacts (STAs) remain an open research question [46]. For these reasons, we do not derive any conclusions on the distractive phase of the motion (negative values of force). We also speculate that the optical system is more susceptible to STAs in deriving force values, due to the fact that the error propagation and the rigid-body assumption for deriving force from positional data are much more complex and uncertain than the process for obtaining force from the inertial data, which is essentially a proportionality. The positional data of several optical markers is used to approximate a rigid body and determine its position, which is then differentiated twice to obtain linear acceleration, while the inertial system directly provides the acceleration itself at the node location, hence making a cleaner measurement, with the strapdown IMU measurement axes directly fixed to local body segment coordinates.

### 3.2. Elbow Valgus Loading and Shoulder Distraction Forces

We have measured two players throwing a series of fastball and change-up pitches (the latter is a slower pitch delivered in a fastball style, meant to confuse batters) with both systems. Table 1 shows the pitch counts and average ball speeds, together with the average peak valgus forces (at the elbow) and the average peak distractive forces (at the shoulder) as derived from the Visual3D analysis described earlier. The means (µ) and standard deviations (σ) of the force estimates are shown for both optical and IMU systems. The ‘factor’, is the ratio between the average peak inferred by the IMU system to the average peak found using the canonically-filtered optical tracker measurements, and indicates the relative scale by which the optical system underestimated the IMU-inferred data. A statistical significance test was performed, given the distribution descriptors and the sample sizes. For the average peak distraction forces, the *p*-values were smaller than 0.0001, indicating that there is a statistically significant difference between the peak measurements for distraction forces on the IMU system in relation to the baseline optical system. For the measurements of the average peak valgus force, only one condition was not statistically significant: player B throwing change-up balls, which had a broader distribution (in general, the small number of change-ups thrown in these tests limit their accuracy, although the means are in line with expected trends). In general, the optical system’s relative underestimate is seen to increase with force. Similar results were seen for shoulder compressive and elbow varus forces [46].

### 3.3. Jerk

**Figure 6:**
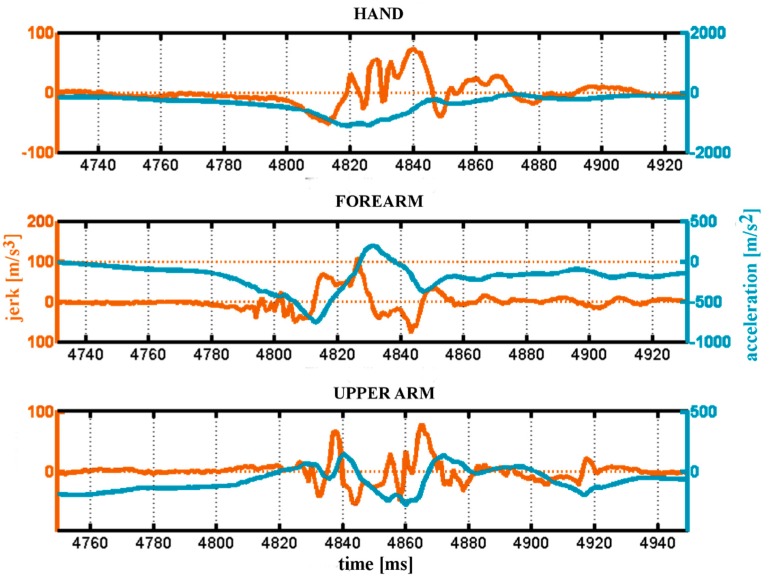
Peaks of acceleration and jerk do not happen at the same moment and have different dynamics.

Our second data collection included data from a separate population of six minor league professional baseball pitchers to investigate the application of jerk as a biomechanical metric for pitch analysis. The linear jerk along the long axis of the humerus (Z_a_-axis) was calculated by directly differentiating accelerometer data from a total of 206 pitches of various pitch types among the six pitchers. We have calculated the average jerk value per pitch type: 2-seam fastball (53.6 m/s^3^), breaking ball (62.3 m/s^3^), change-up (65.0 m/s^3^), fastball (66.8 m/s^3^), and slider (76.3 m/s^3^). In Figure 6, which shows the jerk profile for a typical fastball pitch, one can note that none of the peak jerk values occurred right before or right after the time of peak acceleration. This may be an indicator that analyzing the moment at which the peak jerk occurs could be a relevant assessment for investigating microtrauma that goes beyond just using peak acceleration.

## 4. Discussion

Current optical systems have allowed clinicians to gain insight into the velocities, forces, and torques placed on joints susceptible to injury during ballistic motions and repetitive microtrauma. These estimations, however, are limited by factors such as data filtering methods, lower camera resolution during peak speed of optical camera systems, and artifact motion due to soft tissue movement caused by the high motion speed. Our data is the first to compare a multimodal wearable IMU system to that of an optical system during the ballistic action of the overhand pitch and to investigate the influence of filtering of optical systems compared to IMU data.

Smoothing positional data allows for the use of the optical data for computing kinetic quantities [10,45,57,58,59,60,61]. However, qualitative comparisons of filtered pitching data show loss of potentially meaningful information with the use of standard filters. When looking specifically at valgus elbow torque, our limited dataset demonstrates that calculated optical system elbow torques fall short of those calculated from the inertial system and suggests that stresses on the elbow may be higher than previously evaluated. Some of this limited resolution is related to the capture rate of the systems and the more direct relationship between inertial measurements and forces and torques. The higher capture rate of our sensor-based system (1000 Hz) versus optical systems that commonly capture pitch trials between 240–500 Hz, appears to allow for a better description of the peak dynamics. These results raise an important concern for the field of biomechanics. It is common practice to low-pass filter the optical data to smooth out inherent jitter in the reconstructed optical data. However, the conservative cutoffs used on these filters appear detrimental to the data output, limiting the ability to capture peak dynamics in high-speed athletic motion.

This study also introduces the concept of ‘jerk’ to the evaluation of pitching biomechanics. Jerk, the change in acceleration over time (the 3rd derivative of positional data) cannot be properly derived from optical data of pitching due to the noise within the data, but as shown in this paper, can be usably calculated from IMU data. Measured levels and timing of jerk may offer new assessments of soft tissue injury risk. Evaluation of jerk forces at the shoulder in this study showed a trend toward high jerk forces with sliders compared to fastballs [46], but this did not reach statistical significance with our limited sample of pitchers. A deeper insight into the implications of jerk forces in the shoulder and elbow will benefit from more data collected from such high-rate, wide-range wearable inertial systems.

Clinical speculation is that ligament, tendon, and muscle tissue integrity breaks down due to repetitive microtrauma from repeated high speed pitch deliveries [5,64]. Our preliminary studies have seen indications of frequent changes in acceleration occurring throughout the pitching motion [46]. Although some of this may arise from soft tissue artifacts, such frequent changes in node-measured peak angular velocities indicate accelerations and decelerations not observed in the optical motion data, and may directly contribute to these microtrauma tissue stresses. If this is the case, then the true severity of the trauma is under-measured with use of optical systems. High-speed, wide-range inertial data has the potential to provide both clinician and athlete accurate force/torque information, and in combination with clinical measures/symptoms, offers a unique way to monitor joint stress and joint health.

Although our studies posit a strong argument for the superior veracity of wearable inertial sensors over optical tracking systems for accelerations, forces, torques and parameters inferred from them, one must admit that neither of these systems provide the absolute ground truth. Both systems, camera-based and wearable sensors, measure physical quantities at the skin overlay, and from this data, we are estimating quantities for the underlying rigid bodies, i.e., bones, such as forces and torques. Therefore, there is no noninvasive method today that is able to provide direct, dynamic information of the hidden rigid body structures, especially for fast motion like pitching.

Hence, like all motion analysis studies, this study is limited by soft tissue artifacts (STAs) [46]. For baseball pitching, the ballistic nature of the motion magnifies the effect of STAs. In optical motion-capture systems, STAs are considered the most troublesome source of error [65]. Soft-tissue artifacts manifest in several different ways, such as the inertial reaction of the sensor against elastic skin, and rocking of the sensor as muscles move, contract/extend, and deform underneath during an extreme athletic gesture like pitching [66]. Previous work has shown that upper arm axial rotation (humerus internal-external rotation), is the upper arm motion most affected by STAs [67]. Some studies proposed the mechanical coupling of forearm and upper-arm to compensate for upper-arm artifacts [68]. This technique mitigates the noise in the upper arm data. Even if successful compensation for this coupling is obtained, the solution is limited to motion where the elbow does not reach full extension [68]. This argues that single segment IMU evaluations may not be as accurate as multi-segment models in the future [69].

Wearable sensors offer the ability to collect data across body segments and discover motion patterns that may correlate fatigue with risk of injury to the elbow or shoulder [10]. The inter-segment timing sequence data provided by this system is more accurate than optical systems by nature of the IMU’s high frequency response and rapid synchronized sampling rate. The sensors hence nicely yield the relationship between the timing of each segment’s peak angular velocity, as each transfers its momentum out to the hand during both pitching and bat swinging [46].

Magnetometer-augmented IMU-derived position can be performed when using this system on its own and ‘in the field’, but is not as accurate as position obtained from optical motion capture systems. However, we have seen that the sensors used in the present study provide superior measures of angular velocity, acceleration, and jerk. If one wants to obtain a best set of kinematic and kinetic data, it seems likely that the optimal approach would be to fuse the position and orientation data from a motion capture system with the inertial data obtained from the sensors. One method for fusing the inertial and motion capture data would be to use well-established estimation algorithms based on Bayesian inference that provide a principled way for making optimal inferences from the inertial data and the motion capture system [70]. The use of probabilistic sensor fusion as outlined by Todorov [71] may improve the quality of the joint force and moment estimates, as well as provide the dynamics consistency required for the development of the musculoskeletal models needed to estimate the muscle and ligament forces during a baseball pitch [72].

Using the state-of-the-art camera-based motion capture systems has limitations in data capture rate. The shoulder and elbow distraction torques and forces from such systems are calculated using the second derivative of the measurement system data, i.e., linear acceleration, and inverse kinematics. Unfortunately, the derivatives of orders greater or equal to two have high levels of noise, often resulting in limited or no physical significance, unless the original data—in position units—is filtered down to 10–20 Hz [46]. This filtering damps rapid signal variations, and hampers proper inference of higher-order derivatives that happen during excessive joint load. Accordingly, we assert that classical optical systems do not permit complete insight to the ballistic motion of the baseball pitch, and we hypothesize that a multi-segment inertial system can produce these quantities with increased precision over optical systems.

Our nodes seemed to be mechanically adequate for evaluative scenarios. Skilled motor performers are known to be able to adapt to new setups and interferers around their body easily. The nodes are light and the players did not express concerns related to movement constraints. Most players were queried and there was not a single report of the IMU-based system hindering their performance. The common response indicated that after a few pitches the player “got used to it” and “did not feel it at all”. During our experiments, the players completed their typical bullpen session without complaints for their routine of approximately 50 pitches. We did not design the mechanics of our nodes to be used in actual competitive games, which involve deeper physical and regulatory constraints.

The underlying hardware for wearable IMUs is steadily evolving. The now-common integration of all inertial components onto a single die has been driven by the large mobile devices market, and although this enables a much more compact form factor, such combined devices do not yet provide the extreme dynamic range we need here. The eventual development of log-scale accelerometers and gyros will enable ultra-wide-range, high-resolution devices that do not need redundant measurements at different scales, and will be well suited to measuring athletic gesture in high-intensity sports. Finally, the continual evolution of stretchable electronics is enabling devices such as ours to be embedded in a conformable form factor better suited to mounting on the body [73,74,75], with the caveat that STAs and inertial reaction in intense athletic motion may introduce more effects in a deformable platform.

## Figures and Tables

**Table 1:** Descriptive findings for elbow valgus torque and shoulder distractive force as derived from inertial and optical systems across the two pitchers.

Pitcher	Pitch Type	Sample Size	Average Speed (km/h)	Average Peak Valgus Force (Nm)				Average Peak Distractive Force (N)			
				IMU	Optical	Factor	*p*-value	IMU	Optical	Factor	*p*-value
A	fastball	33	124.4	µ = 159.66 σ = 40.61	µ = 100.22 σ = 7.17	1.59	0	µ = 2994.62 σ = 345.88	µ = 633.29 σ = 38.74	4.73	0
	change-up	3	116.2	µ = 108.76 σ = 1.56	µ = 93.96 σ = 0.73	1.16	0	µ = 2290.87 σ = 228.77	µ = 628.08 σ = 17.69	3.65	0
B	fastball	18	114.9	µ = 75.84 σ = 22.67	µ = 45.39 σ = 6.77	1.67	0	µ = 812.79 σ = 90.90	µ = 519.22 σ = 127.75	1.57	0
	change-up	4	102	µ = 97.57 σ = 20.68	µ = 65.97 σ = 43.76	1.48	0.0871	µ = 794.62 σ = 154.07	µ = 444.90 σ = 63.80	1.79	0
